# Comparative proteomic analysis of the sweetpotato provides insights into response mechanisms to *Fusarium oxysporum* f. sp. *batatas*

**DOI:** 10.1038/s41598-020-78557-y

**Published:** 2020-12-07

**Authors:** ShiQiang Lin, ZhiJian Yang, BiFang Huang, ChuYun Bi, XiaoFang Huang, GuoTai Chen, Nuerla Nijiati, XuanYang Chen

**Affiliations:** 1 Key Laboratory of Crop Biotechnology, Fujian Agriculture and Forestry University, Fujian Province Universities, Fuzhou, China; 2 College of Agriculture, Fujian Agriculture and Forestry University, Fuzhou, China; 3 Key Lab of Genetics, Breeding and Multiple Application of Crops (FAFU), Ministry of Education, Fuzhou, China; 4 College of Life Science, Fujian Agriculture and Forestry University, Fuzhou, China

**Keywords:** Biotechnology, Immunology, Plant sciences

## Abstract

The Fusarium wilt disease caused by *Fusarium oxysporum *f. sp.* batatas* (Fob) is one of the devastating diseases of sweetpotato. However, the molecular mechanisms of sweetpotato response to Fob is poorly understood. In the present study, comparative quantitative proteomic analysis was conducted to investigate the defense mechanisms involved. Two sweetpotato cultivars with differential Fob infection responses were inoculated with Fob spore suspensions and quantitatively analyzed by Tandem Mass Tags (TMT). 2267 proteins were identified and 1897 of them were quantified. There were 817 proteins with quantitative ratios of 1.2-fold change between Fob-inoculated and mock-treated samples. Further, nine differentially expressed proteins were validated by Parallel Reaction Monitoring (PRM). According to Gene Ontology (GO) annotation information, the proteins functioned in molecular metabolism, cellular component formation, and biological processes. Interestingly, the results showed that sweetpotato resistant response to Fob infection included many proteins associated with signaling transduction, plant resistance, chitinase and subtilisin-like protease. The functions and possible roles of those proteins were discussed. The results provides first insight into molecular mechanisms involved in sweetpotato defense responses to Fob.

## Introduction

Sweetpotato (*Ipomoea batatas* L.) is one of the most important food crops in the world and has made great contributions to our food security. The latest statistics showed that in 2017, 9.20 Mha of Sweetpotato were planted in the world with a total production of 12.84 M tons. Currently, sweetpotato is mainly grown in Asia and Africa, In Africa, 4.72 Mha is cultivated with Sweetpotato which surpasses that of Asia 3.97 Mha^1^ .The production of sweetpotato is constrained by numerous biotic and abiotic stresses among which Fusarium wilt is one of the severest. The disease is caused by *Fusarium oxysporum* f. sp. *batatas* (Fob). It is one of the major factors that lead to the general sweetpotato yield loss of 10–50% in southern China^2^. One of the most effective measures of preventing Fusarium wilt is by growing cultivars that are resistant to the disease. Understanding the mechanisms of interaction and co-evolution between the sweetpotato and Fusarium wilt will be advantageous in improving the efficiency of breeding new Fusarium wilt resistant cultivars.

Interactions between crops and pathogens are complex. The gene-for-gene hypothesis proposed by Flor^3^ explains the relationships between crops and pathogenic microorganisms. According to Jones^4^, PAMP-triggered immunity (PTI) and effector-triggered immunity (ETI) are two main mechanisms of crop response to pathogenic microorganisms. The R protein can recognize diverse pathogen effectors directly or indirectly and then deliver the signal downstream by signal transducers such as kinases, transcription factors, phosphatases, and then induce pathogenesis-related proteins, these therefore, enhances serious defense reactions^5^. Many resistance (R) genes were identified and the mechanism of resistance in crops were elucidated by this mode^6,7^.

Recently, the rapid development of molecular biology and bioinformatics, the application of proteomic with high throughput sequencing technologies have enhanced research on protein–protein interaction between crops and pathogens, improving our understanding of the immune system of many crops. Comparative proteomic analysis was conducted to investigate the mechanisms of response to infectious pathogens in a range of crops such as rice, alfalfa, and peanut^8–10^. In sweetpotato, proteomics comparisons were performed to understand the differences between pencil and storage roots, light orange- and purple-fleshed storage roots, contrasting ecotypes, and how the crop responds to drought stress and root-knot nematode^11–15^. However, the differential proteomics of sweetpotato with different resistance response to Fob resistance remains unclear.

In this study, quantitative proteomics were conducted for a comprehensive understanding of the response of sweetpotato to Fob. Tandem mass tags (TMT) combined with UPLC-MS/MS were used with nine interested proteins. The results of this study widens our understanding of the molecular mechanisms of sweetpotato resistance to Fob.

## Results

### Quantitative analysis of sweetpotato defense response to fob

The integrated approach involving TMT labeling and LC–MS/MS was used to quantify the dynamic changes of the whole proteomes in three biological replicates of two sweetpotato cultivars JS57 and XZH. They were exposed to challenged by the *Fusarium oxysporum* f. sp. *batatas* pathogenic strain Fob-07. A total of 2,267 proteins were identified in the three replicates identified and 1,897 were quantified. In order to exploit the potential proteins with special functions related to disease resistance, the fold-change cutoff was set. The proteins with quantitative ratios above 1.2 or below 0.833 are deemed significant. Among the quantified proteins, the amounts of up- or down-regulated proteins in the comparison groups of JS57-07/JS57-CK and XZH-07/XZH-CK in different change folds were similar. Comparisons were also made the comparison between the two comparison groups (JS57-07/JS57-CK) and (XZH-07/XZH-CK) to find out the difference in resistant and susceptible cultivars. The numbers of up- or down-regulated proteins filtered with the threshold value of expression fold change and *p* value < 0.05 were displayed (Table 1).

**Table 1:** Differentially expressed protein in the comparisons (*p* value < 0.05).

Compared groups	Regulated type	Fold change			
		> 1.2	> 1.3	> 1.5	> 2
JS57-07/JS57-CK	Up-regulated	164	136	101	65
	Down-regulated	223	197	127	61
XZH-07/XZH-CK	Up-regulated	168	147	100	65
	Down-regulated	181	157	109	50
JS57-07/JS57-CK vs XZH-07/XZH-CK	Up-regulated	27	19	6	1
	Down-regulated	54	41	14	2

To further understand the functions and features of the identified and quantified proteins, the function or feature of proteins from several different categories, including Gene Ontology, Domain, Pathway, and Subcellular Localizations were annotated. 2267 proteins were identified, quantifiable and annotated. GO annotation information of identified proteins was used to sum up the amount of the differentially expressed proteins in each GO term of level 2. The results showed that the identified proteins covered the range of molecular functions, cellular components and the biological processes. In the molecular functions, the major functional categories identified are binding (1562), catalytic activity (1478), structural molecule activity (173), transporter activity (136) and antioxidant activity (54). In the cellular components, the major categories are cell (1885), organelle (1435), membrane (811), macromolecular complex (514) and symplast (178). In the biological process, the major functional categories identified are cellular process (1607), metabolic process (1600), single-organism process (1289), response to stimulus (564), localization (298), biological regulation (293) and cellular component organization or biogenesis (228).

### Analysis of differential expressed of proteins in sweetpotato response to fob

#### JS57-07 vs JS57-CK

Based on the GO annotation information of identified proteins (Table 2), the amount of the differentially expressed proteins in each GO term of level 2 were summed up. The results showed that a total of 1,086 proteins were up-regulated in the resistant cultivar JS57 in response to Fob07, which belonged to 22 GO terms with the lowest *p* value for 9 biological processes, 9 cellular components and 4 molecular functions. The most enriched GO terms among the 22 GO terms were the catalytic activity (132 proteins), followed by metabolic process (131 proteins), cell (118 proteins), binding (118 proteins), single-organism process (111 proteins) and cellular process (104 proteins). There were 1,563 proteins that were down-regulated and they belonged to 24 GO terms with the lowest *p* value for 10 biological processes, 8 cellular components and 6 molecular functions. The most enriched GO terms among the 24 GO terms were the cell (192 proteins), followed by cellular process (178 proteins), metabolic process (171 proteins), binding (160 proteins), and catalytic activity (154 proteins).

**Table 2:** The numbers of up- and down-regulated proteins of GO distribution.

GO terms level 1	GO terms level 2	JS57-07 vs JS57-CK		XZH-07 vs XZH-CK		JS57-07/JS57-CK vs XZH-07/XZH-CK	
		Up	Down	Up	Down	Up	Down
Biological process	Metabolic process	131	171	134	135	22	43
	Single-organism process	111	138	109	104	17	40
	Cellular process	104	178	103	150	20	38
	Response to stimulus	60	50	52	41	8	16
	Biological regulation	20	31	15	19		6
	Developmental process	12	15		11	2	5
	Multicellular organismal process	11	14			2	
	Localization		28	12	15		5
	Cellular component organization or biogenesis		22	10	18		7
	Multi-organism process	11			11		5
	Other	22	24	19	17	4	7
Cellular component	Cell	118	192	121	156	21	38
	Organelle	92	139	84	114	14	24
	Membrane	39	84	54	59	10	15
	Extracellular region	22	21	32	17	5	10
	Macromolecular complex	17	48	18	38	5	
	Membrane-enclosed lumen	13		13			
	Cell junction	11	19	13	18	4	5
	Symplast	11	19	13	18	4	5
	Other	1	12	1	10	1	5
Molecular function	Binding	118	160	113	128	18	31
	Catalytic activity	132	154	142	115	22	46
	Transporter activity		16	7	6	1	2
	Structural molecule activity		12		14	1	
	Antioxidant activity	14	8	11		2	
	Electron carrier activity			10			
	Other	16	8	5	9		3

KEGG pathway-based enrichment analysis^16^ revealed that 11 pathways with the lowest *p* value were up-regulated in JS57 in response to Fob07, and 5 pathways were down-regulated. The most enriched up-regulated pathways were the Valine, leucine and isoleucine degradation, followed by 2-oxocarboxylic acid metabolism and glycine, serine, and threonine metabolism. There were 5 down-regulated pathways among which amino sugar and nucleotide sugar metabolism were the most enriched ones, followed by photosynthesis and fructose and mannose metabolism.

#### XZH-07 vs XZH-CK

From the GO annotation information of the identified proteins (Table 2), a total of 1091 proteins were found to be up-regulated in the susceptible cultivar XZH in response to Fob07 which belonged to 23 GO terms with the lowest *p* value for 8 biological processes, 9 cellular components and 6 molecular functions. The most enriched GO terms among the 23 GO terms were the catalytic activity (142 proteins), followed by metabolic process (134 proteins), cell (121 proteins) and binding (113 proteins) (Table 2). There were 1,223 proteins down-regulated which belonged to 23 GO terms with the lowest *p* value for 10 biological processes, 8 cellular components and 5 molecular functions. The most enriched GO terms among the 23 GO terms were cell (156 proteins), followed by cellular process (150 proteins), metabolic process (135 proteins), binding (128 proteins), and catalytic activity (115 proteins) (Table 2).

KEGG pathway-based enrichment analysis^16^ revealed that 7 pathways with the lowest *p* value were up-regulated in XZH in response to Fob07, and 5 were down-regulated. The most enriched up-regulated ones were valine, leucine and isoleucine degradation, followed by 2-oxocarboxylic acid metabolism and citrate cycle (TCA cycle). There were 5 down-regulated pathways observed among which porphyrin and chlorophyll metabolism were the most enriched ones, followed by amino sugar and nucleotide sugar metabolism and photosynthesis.

#### JS57-07/JS57-CK vs XZH-07/XZH-CK

In order to find out the differences in response to Fob07 between the two different resistant cultivars JS57 and XZH, the two different proteomics of A (JS57-07 vs JS57-CK) and B (XZH-07 vs XZH-CK) were compared (Table 2). It was found that in the comparisons of A vs B, there were totally 183 up-regulated proteins in total that belonged to 21 GO terms with the lowest *p* value for 10 biological processes, 7 cellular components and 4 molecular functions. The most enriched GO terms among them were catalytic activity (22 proteins) and metabolic process (22 proteins), followed by cell (21 proteins), cellular process (20 proteins), binding (18 proteins), and single-organism process (17 proteins). It was also observed that there were 356 down-regulated proteins which belonged to 21 GO terms with the lowest *p* value of 10 biological processes, 7 cellular components and 4 molecular functions. The most enriched GO terms among the 21 GO terms were catalytic activity (46 proteins), followed by metabolic process (43 proteins), single-organism process (40 proteins), cell (38 proteins) and cellular process (38 proteins) (Table 2).

KEGG pathway-based enrichment analysis^16^ revealed that 3 pathways with the lowest *p* value were up-regulated in A when compared to B, and 5 were down-regulated. The 3 enriched up-regulated pathways were phenylpropanoid biosynthesis, alanine, aspartate and glutamate metabolism, arginine biosynthesis, respectively. The 5 enriched down-regulated pathways were amino sugar and nucleotide sugar metabolism, alanine, aspartate and glutamate metabolism, glycine, serine and threonine metabolism, valine, leucine and isoleucine degradation, ascorbate and aldarate metabolism, respectively.

### Validation by parallel reaction monitoring (PRM) of interested proteins

In order to validated the accuracy of the proteomes using TMT label-based quantitative analysis technique, nine proteins were selected for analysis by PRM (Table 3), Q56YA5 (Serine-glyoxylate aminotransferase), Q6SYB9 (S-adenosylmethionine synthase), P24805 (Stem-specific protein), Q0JF58 (Argonaute 4B), Q9SRH6 (Hypersensitive-induced response protein 3), Q9FYV1 (Inositol-3-phosphate synthase), P13046 (Pathogenesis-related protein R), Q96518 (Peroxidase 16) and O04887 (Pectinesterase 2). The correlation of protein expression levels quantified by TMT and by PRM were compared to examine the accuracy of the data obtained by these two methods. Bivariate correlation analysis was used to calculate the Pearson correlation coefficient among the proteins. The results showed that all of the nine proteins were significantly correlated when both the TMT and by PRM were used to validate the data (r2 > 0.8, *p* < 0.01) (Table 3).

**Table 3:** The correlation of the proteins expression level quantified by TMT and PRM.

	Protein accession	Protein description	Abbr. of gene name	Correlation coefficient(r^2^)	*p* <
1	Q56YA5	Serine–glyoxylate aminotransferase	AGT1	0.915**	0.01
2	Q6SYB9	S-adenosylmethionine synthase	SAMS2	0.987**	0.01
3	P24805	Stem-specific protein TSJT1	TSJT1	0.859**	0.01
4	Q0JF58	Protein argonaute 4B	AGO4B	0.902**	0.01
5	Q9SRH6	Hypersensitive-induced response protein	HIR3	0.963**	0.01
6	Q9FYV1	Inositol-3-phosphate synthase	IPS	0.956**	0.01
7	P13046	Pathogenesis-related protein R	PR	0.921**	0.01
8	Q96518	Peroxidase	PER16	0.921**	0.01
9	O04887	Pectinesterase	PECS	0.944**	0.01

The results obtained (Table 4) showed that the mean expression levels of these proteins were all higher in the cultivars of JS57 and XZH when challenged with Fob-07 than those in the CKs, and the trends were the same when the proteins were quantified by TMT and PRM. However, the range of up-regulated proteins varied for differentially expressed proteins (DEPs), especially for the proteins P13046 and O04887, the ranges quantified by PRM were much higher than those quantified by TMT.

**Table 4:** The range of upregulated proteins quantified by TMT and PRM.

Protein accession	JS57-07/JS57-CK Ratio				XZH-07/XZH-CK Ratio			
	PRM		TMT		PRM		TMT	
Q56YA5	0.36	b A	0.55	a A	0.18	b A	0.35	b A
Q6SYB9	0.13	B c C	0.28	a A	0.09	c C	0.19	b B
P24805	20.25	a A	9.53	a b A	5.60	b A	5.86	b A
Q0JF58	0.19	c C	0.53	a A	0.14	c C	0.30	b B
Q9SRH6	4.55	a A	3.42	a A	4.33	a A	4.44	a A
Q9FYV1	0.12	b B	0.27	a A	0.05	b B	0.21	a A
P13046	5014.96	a A	13.72	b B	6942.06	a A	19.08	b B
Q96518	9.27	a A	8.34	a A	10.15	a A	6.59	a A
O04887	9999.00	a A	10.97	b B	9999.00	a A	14.37	b B

Different lowercase letters among concentrations indicate significant difference(s) at *p* < 0.05, and capital letters indicate disease reaction.

### Identification of the proteins involved in the defense system of sweetpotato response to fob

Sets of proteins that are differentially expressed and directly involved in the immune systems of sweetpotato were differentially expressed was identified (Table 5). Among the eight proteins associated with signaling transduction, three proteins, i.e. Linoleate 9S-lipoxygenase, Auxin-induced protein, Aldo–keto reductase were up-regulated and one protein, i.e. Inositol-3-phosphate synthase was down-regulated in both in the two cultivars. One calcium-binding protein CML13 was down-regulated in the resistant cultivar JS57, while Phospholipase A1 and another Linoleate 9S-lipoxygenase 1 were highly up-regulated extremely. Intriguingly, we found the Aldo–keto reductase 4 was found to be up-regulated in JS57 while it was down-regulated in XZH, suggesting that it may play an important role in the defense response of sweetpotato to Fob.

**Table 5:** The differentially expressed protein in the immune system in sweetpotato invaded by Fob (times).

Function in immune system	Protein accession	Protein description	JS57-07 vs JS57-CK	XZH-07 vs XZH-CK	JS57-07 vs XZH-07	JS57-CK vs XZH-CK	A vs B
Signal transduction	P40691	Auxin-induced protein	2.53	2.15	1.24		
	Q0JE32	Aldo–keto reductase 1	13.31	9.27	2.01		1.44
	Q93ZN2	Aldo–keto reductase 4	2.42	0.30			1.24
	A2ZW16	Phospholipase A1		31.18	0.32		
	Q9FYV1	Inositol-3-phosphate synthase	0.27	0.21			1.30
	Q94AZ4	Calcium-binding protein CML13	0.57				
	P29114	Linoleate lipoxygenase 1	6.49	7.66			
	P27480	Linoleate 9S-lipoxygenase 1		16.95	0.41		
Defense related protein	P13046	Pathogenesis-related protein R	13.72	18.73		1.72	0.73
	P29060	Acidic endochitinase	7.89	3.37			2.34
	P51613	Basic endochitinase	6.33	11.15			0.57
	P93046	Xyloglucan endotransglucosylase	0.49	0.70		1.61	0.71
	Q1PDX5	Subtilisin-like protease SBT3.11			10.34	5.98	
	Q9ZSB0	Subtilisin-like protease SBT3.9			7.79	8.82	
	Q96518	Peroxidase 16	8.25	6.39			1.29
	Q9FKA4	Peroxidase 62	5.97	4.08			1.46
	Q9FLC0	Peroxidase 52	4.81	5.39			
	P86001	Peroxidase 3	4.43	3.76	1.40		
	Q01297	Catalase isozyme 1	1.96		1.31	0.55	
	P85076	Pectinesterase 1	0.43		4.49	10.82	
	O04887	Pectinesterase 2	10.43				
	Q9SRH6	Hypersensitive-induced response protein 3	3.41	4.31			0.79
	Q7XZR1	S-adenosylmethionine synthase 1	0.12	0.15			
	Q6SYB9	S-adenosylmethionine synthase 2	0.28	0.19			1.49
	P42735	Cadmium-induced protein	6.91				

A number of defense-related proteins differentially regulated were screened, they include the PR, chitinase, Subtilisin-like protease, Peroxidase, Pectinesterase and so on. Most of them were found to be up-regulated in both the two cultivars. Five were found to be up- or down-regulated in JS57 only. Interestingly, two subtilisin-like proteases were found not to be up- or down-regulated after exposed to Fob, but the levels were higher in JS57 than those of XZH which implies the subtilisin-like protease may contribute to the defense system acting as PTI.

## Discussion

One of the most effective ways to control plant diseases is by breeding resistant cultivars for use in production and cultivation. Understanding the mechanisms of plant’s resistance to diseases and identifying resistance genes helps to promote the breeding process. There exists a complicated interactions between crops and pathogens. The crops recognize invading pathogens by receptors and trigger defense responses by activating signaling transduction through MAP kinases (MAPKs), oxidative burst, ion influx which induces expression of defense-related genes^17^.

In this study, the quantitative proteomics was used and a number of defense-related proteins were identified in sweetpotato and how they respond to Fob. Some proteins were found to be associated with signal transduction and were differentially expressed between the resistant cultivar JS57 and the susceptible cultivar XZH, which may contribute to their defense response. It was also discovered that there were two aldo–keto reductases which may have different roles in the response of sweetpotato to Fob infection:the aldo–keto reductases 1 was up-regulated both in JS57 and XZH, and the aldo–keto reductases 4, was up-regulated in JS57 while down-regulated in XZH. The aldo–keto reductases belong to a superfamily that contains more than 190 members associated with carbonyl substrates reduction in all phyla^18^. In plants the aldo–keto reductase may enhance tolerance to various abiotic stresses. One of the aldo–keto reductase genes from sweetpotato was found to transfer high tolerance of cadmium stress in tobacco^19^. In the current study, it was discovered that aldo–keto reductases also contributed to Sweetpotato tolerance to biotic stress (Fob) and the aldo–keto reductases 4 may have special roles since it was differentially regulated in JS57 and XZH.

Mitogen-activated protein kinases (MAPKs) are important signal transducing enzymes that are involved in many aspects of cellular regulations^20^. Phospholipases may be activated in response to various cellular and environmental cues and affect various cellular processes through their roles in signal transduction. In this study, Phospholipase A1 was fond to be highly up-regulated (31.18 times) in the susceptible cultivar XZH’s response to Fob only. This explains its important role in conferring resistance. Thus, the Phospholipase A1 needs to be better understood than other members of the family such as Phospholipase A2, D, C and B^20^.

Pectins are the major components of plant’s cell walls and play a key role in forming the first defense barrier against pathogen colonization. The degree of esterification of pectin, determined by Pectinesterase, affects the structure and properties of the cell wall which is involved in resistance to pathogens^21^. The abundance of fully de-esterified, low and partially methylesterified HGs Pectinesterase is related to pathogen susceptibility^22^. Overexpression of pectin methyl esterases inhibitor proteins reduces the activity or the expression level of pectin mechyl esterification resulting in higher resistance to pathogens^23^. Pectinesterase 1 was found to be down-regulated in resistant cultivar JS57’s response to Fob, which may have resulted from the structural stability of pectin in the sweetpotato’s cell walls. Intriguingly, we found another Pectinesterase 2 located in the vacuolar membrane was found to be up-regulated by 10.43 times in JS57. The levels in expressions of both Pectinesterases were unchanged in the susceptible cultivar XZH in its response to Fob. These results demonstrated that pectinesterases plays important roles in plant-pathogen interactions but the way each member of the family works is different.

It was reported that S-adenosylmethionine synthase (SAMs) catalyzes the biosynthesis of S-adenosylmethionine which serves as the methyl donor for pectin methylesterase^24^ and the precursor for the biosynthesis of ethylene^25^. This shows that SAMs is very essential to the biotic stress response of plants, as the pectin is one of the components of cell wall and ethylene is a key molecule in signal transduction.

Subtilisin-like proteases are a large family of enzymes consisting of many diverse members in plants with various biological functions including protein turnover, plant development and interactions with the environment. Recently, subtilases were found to be involved in pathogen resistance and plant immunity^26^. In this study, two subtilisin-like proteases of the subfamily 3, SBT3.11 and SBT3.9, with higher expressions in JS57 than in XZH were identified. However, protease was not found to increase in the response to Fob, and did not show any difference in the two cultivars. These indicated that they may play a significant role in the PTI.

From the current research, sets of proteins that are involved in the defense systems of sweetpotato to Fob were identified, their functions of which need to be further exploited. Previously, the same cultivars (JS57 than XZH) and pathogens (Fob) were used to identify disease resistance genes in sweetpotato by performing de novo transcriptome assembly and digital gene expression analysis^27^, but there were few identical genes/proteins found in the study.

## Materials and methods

### Plant and pathogen materials, treatment

Two sweetpotato cultivars, JS57, with high resistance, and XZH, highly susceptible to Fusarium wilt, were used as materials. These cultivars were the ones used to investigate the digital gene expression response against *Fusarium oxysporum* f. sp. *batatas *^27^.

The seedlings of the two cultivars were grown in the field for 4 weeks and then freshly excised into a length of about 15 cm and prepared for subsequent treatment.

The pathogen strain F07 of *Fusarium oxysporum* f. sp. *batatas* was activated by growing on potato-saccharose-agar (PSA) medium in a Petri dish at 28 °C for about 1 weeks, and then moved into a new Petri dish with fresh PSA until the mycelia covered the surface of the medium. The spores were collected and prepared into conidia solutions of 1 × 10^7^ conidia/mL in glass bottles, as described by Yang^20^. The sweetpotato seedlings were inoculated into the bottles with conidia solutions and water as a control and kept in growth chambers at 28 °C for 24 h. Then the basic stems with a length of 2 cm were excised as samples and immediately kept frozen in liquid nitrogen. The experiment was composed of three independent biological replicates with four treatments as follows: JS57 cultured in the Fob solution (JS57-07), JS57 cultured in water (JS57-CK), XZH cultured in the Fob solution (XZH-07), and XZH cultured in water (XZH-CK).

### Protein isolation, digestion and TMT labeling

The seedling samples were grounded for 30 min in liquid nitrogen and then sonicated three times using a high intensity ultrasonic processor (Scientz) on ice in lysis buffer (8 M urea, 2 mM EDTA, 10 mM DTT and 1% Protease Inhibitor Cocktail). After the samples were centrifuged at 20,000×*g* at 4 °C for 10 min, the remaining debris was removed, and the protein was precipitated with exposure to 15% TCA for 4 h at − 20 °C. The supernatant was discarded after centrifugation at 4 °C for 3 min. The proteins were rinsed three times with cold acetone and then re-dissolved in a buffer and the concentration determined with a 2-D Quant kit following the manufacturer’s guide^28^.

For the digestion, the proteins solution were reduced with a 5 mM DTT for 30 min at 56 °C and alkylated with 11 mM IAA for 15 min at room temperature in darkness. For trypsin digestion, the proteins samples were diluted by adding 200 mM TEAB to urea concentration (less than 2 M). The trypsin was added at a ratio of 1:50 trypsin-to-protein mass ratio for the first digestion overnight and 1:100 trypsin-to-protein mass ratio for the second 4 h-digestion. Approximately 100 μg protein from each of the samples was digested with trypsin for the subsequent experiments^28^.

After trypsin digestion, peptide was desalted by Strata X C18 SPE column (Phenomenex) and vacuum-dried. It was reconstituted in 1 M TEAB and processed according to the manufacturer’s protocol for 6-plex TMT kit. Briefly, one unit of TMT reagent (defined as the amount of reagent required to label 100 μg of protein) was thawed and reconstituted in 24 μl ACN. The peptide mixtures were then incubated for 2 h at room temperature and pooled, desalted and dried by vacuum centrifugation^28^.

### HPLC fractionation and LC–MS/MS analysis

The samples were then fractionated into fractions by high pH reverse-phase HPLC using Agilent 300Extend C18 column (5 μm particles, 4.6 mm ID, 250 mm length). Peptides were first separated briefly with a gradient of 2% to 60% acetonitrile in 10 mM ammonium bicarbonate pH 9.0 over 80 min into 80 fractions. Then, the peptides were combined into 18 fractions and dried by vacuum centrifugation^28^.

The Peptides were dissolved in 0.1% FA and directly loaded onto a reversed-phase pre-column (Acclaim PepMap 100, Thermo Fisher Scientific). Peptide separation was performed using a reversed-phase analytical column (Acclaim PepMap RSLC, Thermo Fisher Scientific). The gradient was composed of an increase from 5 to 25% solvent B (0.1% FA in 98% ACN) over 26 min, 25% to 40% in 8 min and climbing to 80% in 3 min then holding at 80% for the last 3 min, all at a constant flow rate of 350 nL/min on an EASY-nLC 1000 UPLC system^28^.

The peptides were subjected to NSI source followed by a tandem mass spectrometry (MS/MS) in Q Exactive (Thermo Fisher Scientific) coupled online to the UPLC. The intact peptides were detected in the orbitrap at a resolution of 70,000. Peptides were selected for MS/MS using NCE setting as 28; ion fragments were detected in the orbitrap at a resolution of 17,500. A data-dependent procedure that alternates between one MS scan followed by 20 MS/MS scans was applied for the top 20 precursor ions above a threshold ion count of 1E4 in the MS survey scan with 30.0 s dynamic exclusion. The electrospray voltage applied was 2.0 kV. Automatic gain control (AGC) was used to prevent overfilling of the orbitrap; 5E4 ions were accumulated for generation of MS/MS spectra. For MS scans, the m/z scan range was 350 to 1800. Fixed first mass was set at 100 m/z^28^.

### Database search

The resulting MS/MS data were processed using MaxQuant with integrated Andromeda search engine (v.1.5.2.8). Tandem mass spectra were searched against SwissProt Green Plant database. Trypsin/P was specified as cleavage enzyme allowing up to 2 missing cleavages. Mass error was set at10 ppm for precursor ions and 0.02 Da for fragment ions. Carbamidomethyl on Cys, was specified as fixed modification and oxidation on Met and acetylation on protein N-term was specified as variable modifications. For the protein quantification method, TMT 6-plex was selected in Mascot. FDR was adjusted to < 1% at protein, peptide and PSM level^29^.

### Validation by parallel reaction monitoring (PRM)

Nine proteins Q56YA5 (serine-glyoxylate aminotransferase), Q6SYB9 (S-adenosylmethionine synthase), P24805 (stem-specific protein), Q0JF58 (argonaute 4B), Q9SRH6 (hypersensitive-induced response protein 3), Q9FYV1 (inositol-3-phosphate synthase), P13046 (pathogenesis-related protein R), Q96518 (peroxidase 16), O04887 (pectinesterase 2) were selected for validation by Parallel Reaction Monitoring (PRM). The proteins of the sample were extracted and then digested by trypsin. The tryptic peptides were dissolved in 0.1% formic acid (solvent A), directly loaded onto a home-made reversed-phase analytical column (15-cm length, 75 μm i.d.). The gradient was composed of an increase from 6 to 23% solvent B (0.1% formic acid in 98% acetonitrile) over 38 min, 23% to 35% in 14 min and climbing to 80% in 4 min then holding at 80% for the last 4 min, all at a constant flow rate of 400 nL/min on an EASY-nLC 1000 UPLC system^29^.

The peptides were subjected to NSI source followed by tandem mass spectrometry (MS/MS) in Q Exactive Plus (Thermo Fisher Scientific) coupled online to the UPLC. The electrospray voltage applied was 2.0 kV. The m/z scan range was 350 to 1000 for full scan, and intact peptides were detected in the Orbitrap at a resolution of 35,000. Peptides were then selected for MS/MS using NCE settings at 27 and the fragments were detected in the Orbitrap at a resolution of 17,500. A data-independent procedure that alternated between one MS scan followed by 20 MS/MS scans. Automatic gain control (AGC) was fixed at 3E6 for full MS and 1E5 for MS/MS. The maximum IT was set at 20 ms for full MS and auto for MS/MS. The isolation window for MS/MS was set at 2.0 m/z^29^.

The generated MS data were processed using Skyline (v.3.6). Peptide settings: enzyme was set as Trypsin [KR/P], Max missed cleavage set at 2. The peptide length was set at 8–25, Variable modification was set as Carbamidomethyl on Cys and oxidation on Met, and max variable modifications were set at 3. Transition settings: precursor charges were set as 2, 3, ion charges were set at 1, 2 and ion types were set as b, y, p. The productions were set as from ion 3 to last ion, the ion match tolerance was set at 0.02 Da^29^.
